# The Impact of Delayed Surgical Care on Patient Outcomes With Alimentary Tract Perforation: Insight From a Low-Middle Income Country

**DOI:** 10.7759/cureus.27592

**Published:** 2022-08-02

**Authors:** Muhammad H Zafar, Taha A Zaka Ur Rehman, Muhammad Sohaib Khan, Shayan Ahmed, Amir Shariff

**Affiliations:** 1 Surgery, Aga Khan University Hospital, Karachi, PAK

**Keywords:** surgical care, emergency laparotomy, global surgery, trauma, alimentary tract perforation

## Abstract

Introduction

In-patient delay is associated with increased mortality in patients with alimentary tract perforations. Access to surgical care is a glaring health issue in low-middle income countries (LMICs), where patient presentation is also delayed for a myriad of reasons, which can be broadly categorized as social/cultural, financial, and structural in their nature. The impact these delays have on surgical outcomes in low-middle income countries is not known.

Methods

A retrospective cohort study of patients who underwent emergency laparotomy for alimentary tract perforation from July 2015 to June 2018 was conducted at a tertiary care hospital in Karachi, Pakistan. Time was recorded in two variables: symptom onset to emergency room presentation (ERT) and emergency room to operation room time (ORT).

Results

Overall, 80 patients were included in the study. The 12 (15%) patients who expired were significantly older (57 ± 17.7 years of age), had a higher Charlson Comorbidity Index and had longer ORT [median ORT in hours-discharged vs expired: 8.2 (IQR 5-15) vs 16 (IQR 12-28) p=0.02]. ERT was also longer but lacked statistical significance [median ERT in hours-discharged vs expired: 24 (IQR 22-72) vs 48 (IQR 24-120) p=0.19]. Multivariable logistic regression analysis revealed ORT to be significantly associated with mortality [odds ratio (OR): 1.02, 95% confidence interval (CI): 1.003-1.041; p=0.02]. Adjusted Cox regression analysis showed that each hour of ORT increased the risk of mortality by 1.5% [hazard ratio (HR) 1.015, 95% CI 1.001-1.030].

Conclusion

Inpatient delays increased the risk of mortality for patients undergoing emergency laparotomy for alimentary tract perforation. Larger sample sizes and prospective studies are needed to better understand this relationship and the impact pre-hospital delays have on outcomes.

## Introduction

The alimentary tract runs from the mouth to the anus. Alimentary tract perforation usually involves the stomach, duodenum, small intestine, or large bowel and occurs as a result of full-thickness gastrointestinal wall injury [1]. It is associated with high morbidity and mortality and therefore requires immediate surgery [2-5]. The global burden and the financial implications are enormous [6]. The most affected are resource-limited countries, where mortality can be as high as 80% [4]. In some of these regions, an estimated 95% of the population lacks access to timely surgical care [7]. While the correlation can be extrapolated, the impact this lack of access has on surgical outcomes of alimentary tract perforation is largely unknown. Even in developed countries where most patients have access to surgical care, the mortality from this condition can be high [2]. This stems from the severity of the condition and the complex interplay of various factors that impact the outcome [2,3]. Factors such as advanced age, multiple comorbidities, malignancy, and nutritional deficiencies are related to the patient's baseline condition and thus cannot be addressed.

Emergency room length of stay and pre-operative delays are complex problems with important implications. It reflects the increasing demands on the system and organizational deficiencies as well as the complexities of patient management related to the severity of the condition [8-10]. Evidence suggests that inpatient pre-operative delay is associated with an adverse outcome in patients with alimentary tract perforation [11-13]. Other studies have refuted these findings [14]. Moreover, a very limited number of studies are from low-middle income countries (LMICs), where patient and hospital-related factors are different. In one such systematic review which aimed to characterize the barriers to accessing health care in developing countries, Irfan et al. categorized these reasons as being at the level of the patient, environment, health system, and the provider [15]. Patient-level factors which hindered access to seeking timely surgical care, such as regional attitudes and societal norms, and the prevalence/local support for practitioners of alternative medicine described in the review, were strikingly different from those observed in the western world.

In light of the recent literature on the limitations of access to surgical care globally, the question of the impact of time taken for presentation and then subsequent surgery on outcomes has immense significance. In this study, we aimed to determine how delays in surgical care impact mortality in patients with alimentary tract perforation.

An abstract for this paper was presented at the American College of Surgeons Clinical Congress in 2019, which has been published in the Journal of the American College of Surgeons.

## Materials and methods

This was a retrospective cohort study of patients who underwent emergency laparotomy for alimentary tract perforation from July 2015 to June 2018. It was conducted at a tertiary care hospital in Karachi, Pakistan after receiving approval from the institutional ethical review committee. Time was recorded in two variables: symptom onset to emergency room presentation (ERT) and emergency room to operation room time (ORT). Potentially confounding variables that were assessed as being present at the onset of symptoms were collected. These included the patients’ demographics and health variables, namely age, gender, height, weight, body mass index, and comorbidities. These variables were used to determine the Charlson Comorbidity Index for each patient. Surgical variables based on intraoperative findings, namely the site of perforation, presence of malignancy, and feculent ascites, were also included.

Stata/IC 15.1 was used for analysis. P-values of less than 0.05 were considered significant. Descriptive analyses were performed using the Chi-square and independent samples T-test for categorical and continuous variables, respectively. Categorical variables are described with frequencies and percentages. Continuous variables are described as either mean ± standard deviation when normally distributed or median with inter quartile range when not normally distributed, using the Mann-Whitney U test. All variables with P-values less than 0.2 were adjusted for in the final analysis. Multivariable logistic regression and Cox regression models were used to test for a significant association between time to surgical care and mortality.

## Results

Overall, 80 patients were included in the study. Of these, 12 (15%) patients died during their hospital stay while 68 (85%) were discharged. The two groups were similar in their gender distribution and mean body mass index. Patients who had expired were found to be significantly older with a mean age of 57 (vs. 42.7 years old for patients who were discharged, p=0.02) and had a higher Charlson Comorbidity Index (p=0.01) (Table 1).

**Table 1:** Patient demographics SD: standard deviation

Variables	Discharged (n=68)	Expired (n=12)	p-Value
Mean age ± SD (years)	42.7 ± 20.6	57 ± 17.7	0.02
Females (%)	21 (31)	4 (33)	0.86
Mean height ± SD (centimeters)	164 ± 7.8	162 ± 4.4	0.40
Mean weight ± SD (kilogram)	68.8 ± 12.6	65.4 ± 20.3	0.40
Mean body mass index ± SD	25.7 ± 4.8	25.3 ± 8.3	0.80
Charlson Comorbidity Index			
Score 0-2	52 (76.5)	4 (33.3)	0.01
Score 3	4 (5.9)	2 (16.7)	
Score 4	6 (8.8)	2 (16.7)	
Score 5	2 (2.9)	3 (25)	
Score ≥6	4 (5.9)	1 (8.3)	

As for the intraoperative variables collected, there was a trend for discharged patients to have a duodenal perforation (47%), while expired patients were more likely to have an ileal perforation (41.7%), but this difference did not reach statistical significance (p=0.5). Moreover, no difference was observed between the proportion of patients who were found to have malignant or feculent ascites in the two groups. However, patients who died had a longer ORT [median ORT in hours - discharged vs expired: 8.2 (IQR 5-15) vs 16 (IQR 12-28) p=0.02]. ERT was also longer but lacked statistical significance [median ERT in hours - discharged vs expired: 24 (IQR 22-72) vs 48 (IQR 24-120) p=0.19] (Table 2).

**Table 2:** Patient characteristics IQR: interquartile range

Variables		Discharged (n=68)	Expired (n=12)	p-value
Time to emergency room presentation in hours (IQR)		24 (22-72)	48 (24-120)	0.19
Time to operation room in hours (IQR)		8.2 (5-15)	16 (12-28.5)	<0.01
Site of perforation (%)				0.50
Duodenum		32 (47)	3 (8.6)	
Ileum		20 (29.4)	5 (41.7)	
Appendiceal		5 (7.4)	1 (16.7)	
Colorectal		11 (16)	3 (25)	
Malignant perforation	Present	8 (11)	2 (16)	0.60
	Not present	60 (88)	10 (83)	
Feculent ascites	Present	11 (16)	3 (25)	0.40
	Not present	57 (83)	9 (75)	

Multivariable logistic regression analysis revealed ORT to be significantly associated with mortality [odds ratio (OR): 1.02, 95% confidence interval (CI): 1.003-1.041; p=0.02] (Table 3).

**Table 3:** Multivariable logistic regression analysis OR: odds ratio, CI: confidence interval

Variables	OR	p-Value	95% CI	
Age	1.03	0.12	0.99	1.08
Charlson Comorbidity Index	1.21	0.50	0.68	2.15
Time to emergency room presentation in hours	1.00	0.20	0.99	1.01
Time to operation room in hours	1.02	0.02	1.00	1.04

Adjusted Cox regression analysis showed that each hour of ORT increased the risk of mortality by 1.5% [hazard ratio (HR) 1.015, 95% CI 1.001-1.030] (Table 4).

**Table 4:** Multivariable Cox regression analysis HR: hazard ratio, CI: confidence interval

Variables	HR	p-Value	95% CI	
Age	1.03	0.15	0.99	1.08
Charlson Comorbidity Index	1.32	0.26	0.82	2.13
Time to emergency room presentation in hours	1.00	0.26	1.00	1.01
Time to operation room in hours	1.02	0.04	1.00	1.03

## Discussion

In our study, inpatient delay was associated with increased mortality. However, delays in the patient's presentation did not significantly impact the outcome. This is one of the few studies in emergency general surgery that has attempted to understand the relationship between both the delays in presentation and preoperative management and mortality in patients with alimentary tract perforation. This is in contrast to obstetrical and maternal care, where the three-delayed model introduced over two decades ago is now well established. In this model, delays occurred in three distinct steps: the decision to seek care, the process to reach care, and then, subsequently receiving care at the health care facility [16]. Through numerous studies, the factors involved in each of the three delays in maternal care have been well studied [17-19]. For this reason, the three-delay model provided the basis for the Lancet commission for outlining and addressing the challenges of healthcare delivery and management in global surgery [20]. However, the factors involved in these delays and their impact on outcomes in other surgical disease processes have not been extensively studied and are especially lacking in low and low-middle income countries.

Perforation of the alimentary tract leads to shock, peritonitis, and sepsis, which result in high mortality [5,2]. Source control via emergency surgery is vital for survival [21,22]. Since early initiation of resuscitation and placement of drains with normal saline lavage through the drains in the management of sepsis is recommended owing to better survival [22], it should follow that early surgery for source control from the onset of symptoms should also lead to better survival. However, this cannot be reliably substantiated with the level and quality of evidence at present. Patients in low and LMICs are more likely to encounter pre-hospital delays, but since clinical research output from these regions is limited, the impact of these delays is not known [23]. The factors leading to inpatient delays would vary between regions and countries. However, the clinical basis for optimal timing of intervention would be similar globally. Even then, data to support any recommendation in this regard is generally weak [21].

The time from the onset of symptoms to a presentation at the emergency department encompasses the first two of the three delays model, i.e., time spent in deciding about seeking care and then reaching the health care facility. This is reflected in the duration of presenting complaints noted at the first point of contact with the healthcare provider. The impact this time duration has on the outcomes of patients with alimentary tract emergencies is infrequently studied and inconsistently reported. In recently published studies [24,25], complicated appendicitis has been found to be associated with a longer duration of symptoms. Delays in the presentation of patients with ileal perforation have also been found to be associated with increased mortality [26]. However, considering the fact that globally, 5 billion people lack access to essential surgical care [7], the studies that have determined the impact of delays in presentation from low and low-middle-income countries are almost negligible. In our study, patients who expired presented at a median of 48 hours after the onset of symptoms. However, to our surprise, the difference in this pre-hospital delay among those who survived was not statistically significant.

The Surgical Infection Society recommends that source control procedures for complicated intra-abdominal infections should be carried out within 24 hours of admission and earlier for sick patients [21]. Other studies have shown improved survival for patients who underwent source control at an earlier time [11,13]. Unchecked, all septic patients would worsen over time. After admission to the emergency room, time is unlikely to be the lone determinant for improved survival; rather, it would be the interventions and clinical endpoints that are either achieved or delayed during a given time period that would impact the outcome. These have not been elucidated. The observation of the impact of inpatient delay has on outcomes does, however, reflect their importance. In our study, inpatient delay was observed to be significantly associated with mortality while delayed presentation or pre-hospital delay was not. One possible inference from this observation could be that faster inpatient care conferred a more significant survival advantage than any disadvantage conferred by pre-hospital delay.

This study, even though demonstrates significant findings, does have limitations. Variables that would reflect the severity of sepsis have not been included in the analysis. These data may have shed light on the potential causes of the inpatient delay to surgery, with it being rationalized that patients with more advanced sepsis would require more time for resuscitation and stabilization prior to operative intervention. However, adjusting for the severity of the patient’s condition would have nullified the impact of pre-hospital delay which was the primary objective of our study. Therefore, only the baseline variables were included as confounders. Furthermore, since this is a single center study from one of the most highly established urban health care center of the country, it cannot reflect the delays and outcomes faced by patients in semi-urban and rural settings.

## Conclusions

In this study, inpatient delays had a more significant impact on mortality for patients undergoing emergency laparotomy for alimentary tract perforation than outpatient prehospital delays. Reducing preoperative delays by optimizing inpatient care has the potential to significantly reduce mortality. Studies conducted in rural settings with a larger sample size are needed to better understand the impact of outpatient prehospital delays on patient outcomes.
